# The Health Benefits of *Tamarindus indica*: A Focus on the Relationship Between Phytochemical Composition and Physiological Effects

**DOI:** 10.3390/nu18040576

**Published:** 2026-02-09

**Authors:** Carlos Rabeiro Martinez, Yasmany Armas Diaz, Danila Cianciosi, Qingwei Cao, Haixia Hu, Ge Chen, Zexiu Qi, Bei Yang, José L. Quiles, Maurizio Battino, Francesca Giampieri

**Affiliations:** 1 Department of Clinical Sciences, Università Politecnica delle Marche, 60131 Ancona, Italy; c.l.rabeiro@pm.univpm.it (C.R.M.); y.armas@pm.univpm.it (Y.A.D.); d.cianciosi@staff.univpm.it (D.C.); q.cao@pm.univpm.it (Q.C.); h.hu@pm.univpm.it (H.H.); g.chen@pm.univpm.it (G.C.); z.qi@pm.univpm.it (Z.Q.); b.yang@pm.univpm.it (B.Y.); f.giampieri@univpm.it (F.G.); 2 Joint Laboratory on Food Science, Nutrition, and Intelligent Processing of Foods, Polytechnic University of Marche, Italy, Universidad Europea del Atlántico Spain and Jiangsu University, China at the Polytechnic University of Marche, 60131 Ancona, Italy; 3 Department of Physiology, Institute of Nutrition and Food Technology “José Mataix Verdú”, Biomedical Research Centre, University of Granada, 18100 Armilla, Spain; jlquiles@ugr.es; 4 Research Group on Food, Nutritional Biochemistry and Health, Universidad Europea del Atlántico, Isabel Torres 21, 39011 Santander, Spain; 5 International Joint Research Laboratory of Intelligent Agriculture and Agri-Products Processing, Jiangsu University, Zhenjiang 212013, China

**Keywords:** *Tamarindus indica*, antioxidants, phenolic compounds, inflammation, cardio-protection, antidiabetic, antibacterial, hepatoprotection

## Abstract

**Background/Objectives**: Conventional pharmacotherapy for the most prevalent human diseases still has limited efficacy. Natural medicines are recognized for their therapeutic efficacy and low side effects. *Tamarindus indica* is a tropical tree of the *Fabaceae* family, valued for its multiple uses and the nutritional properties of its fruits. The purpose of this review is to provide an overview of the nutraceutical value of *T. indica*, focusing on its phytochemical composition and main health benefits. **Methods**: For this purpose, a bibliography search was performed in PubMed, Scopus, and ScienceDirect databases, including all articles published between 2000 and December 2025. **Results**: The *T. indica* fruit contains different phytochemical compounds, such as flavonoids, tannins, alkaloids, and saponins, with therapeutic potential. These compounds exert free radical scavenging activity, improve antioxidant and detoxification enzyme activities, exert antimicrobial effects, attenuate the activation of pro-inflammatory mediators, and regulate the expression of lipid metabolism genes. **Conclusions**: This article presents an integrated analysis summarizing the phytochemical characteristics, mode of action, medical utility, and safe use of *T. indica*, thereby contributing to a greater understanding of its potential health benefits.

## 1. Introduction

Natural therapies have demonstrated positive effects in many different human pathologies, such as diabetes, cancer, cardiovascular diseases (CVDs), and infectious diseases [1]. When these therapies are applied at suitable dosages and for appropriate periods, according to the context and necessities of each case, they act as cytoprotectors, immunomodulators, or antimicrobials [2,3]. The World Health Organization (WHO) recognizes that herbal medicine represents an efficient strategy to counteract many health conditions [4], with lower toxicity, fewer medical interactions, and greater accessibility than many conventional drugs. Given that, in many cases, natural therapies form part of the diet, health organizations are calling for their implementation [5,6]. Some natural foods with demonstrated health properties include curcuma [7], strawberry [8], garlic [9], elderberry [10], mangoes [11], and sea buckthorn [12].

**Figure 1:**
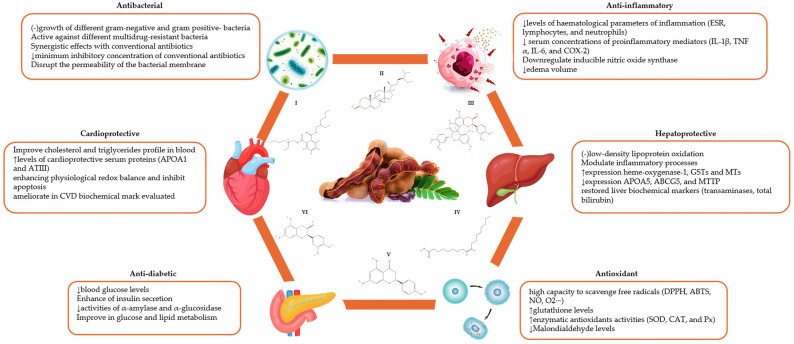
Summary of the health effects of *Tamarindus indica* in in vitro and in vivo studies. ↓: reduction, ↑: increase, -: inhibition, CVD: cardiovascular diseases, IL: interleukin, TNF α: tumor necrosis factor-alpha, DPPH: 2,2-diphenyl-1-picrylhydrazyl, ABTS: 2,2′-azino-bis(3-ethylbenzothiazoline-6-sulfonic acid), APOA1: apolipoprotein A1, ATIII: antithrombin III, GSTs: glutathione S-transferases, MTs: metallothioneins, SOD: superoxide dismutase, CAT: catalase, Px: peroxidase, ESR: erythrocyte sedimentation rate, NO: oxide nitric, O2∙−: superoxide anion radical, APOA5: apolipoprotein A5, MTTP: microsomal triglyceride transfer protein, I: bis (2-ethylhexyl) phthalate, II: gamma-sitosterol, III: procyanidin B2, IV: oleic acid, V: naringenin, VI: epicatechin.

*Tamarindus indica* L. is a multipurpose tree most appreciated due to its culinary and traditional medical use [13]. Different studies have identified that *T. indica* has compounds with antimicrobial [14], antioxidant [15], anti-inflammatory [16,17], cardioprotective [18], antihyperlipidemic, and hepatoprotective [19] properties. The purpose of the present review is to summarize the current knowledge on the nutraceutical potential of *T. indica* in addition to its phytochemical composition and evidenced health benefits (Figure 1).

A systematic search was performed within the PubMed, ScienceDirect, and Scopus databases using the words *T. indica* or tamarind together with other specific keywords such as phytochemical, antioxidants, phenolic compounds, inflammation, cardio-protection, antidiabetic, antibacterial, hepatoprotection, and toxicological study. The literature search included all articles (review articles, original papers, meta-analyses, and book chapters) published in the English language from 2000 up to December 2025. Articles without full text available or articles that were not relevant were excluded. In addition, the chemical structures were taken from the PubChem database.

## 2. General Composition

*Tamarindus indica* L. is a fruit tree member of the family *Fabaceae* (*Leguminosae*). *Tamarindus* is a monotypic genus with a sole species (*T. indica*). It is a long-lived and evergreen tree, around 20–25 m tall. According to consensus, *T. indica* is native to the Eastern African region, but currently it is distributed in the tropical regions around the world [13]. Some countries in Southeast Asia and America produce *T. indica* as a commercial crop, with Asian countries such as India being the main producers [20]. In the international market, *T. indica* production is mainly destined for the food industry, while the pharmaceutical industry also uses it for the manufacture of excipients [21].

*T. indica* fruit is the most valuable part of the tree, due to its culinary, medicinal, and industrial applications [22]. The mature fruits are ash-brown pods, 5 to 16 cm long and around 2 cm broad [15]. These fruits are formed by shell and fiber (11–30%), pulp (30–50%), and seeds (25–40%). The pulp of the fruit is often consumed raw or processed for use in various culinary preparations, such as juices, jams, pickles, and spices [23]; its seeds are roasted and consumed as snacks. Furthermore, the by-products (shells, fibers and seeds) generated during the industrial process of obtaining the *T. indica* fruit pulp are also utilized in food and non-food production [24,25]. The immature fruit, flowers, and leaves are used in a few countries to make stews, curries, soups, and salads, or as part of traditional medicine [26].

### Tamarindus indica: Fruit and Seeds

The nutritional composition of *T. indica* pulp is characterized by its high concentration of carbohydrates, dietary fiber (beneficial in digestive health), and proteins (Table 1) [27,28].

Additionally, it is rich in essential minerals such as calcium, magnesium, phosphorus, and potassium (Table 2) [27,28]. These values can vary depending on the climate and soil characteristics, as well as the phenological steps of the tree, fructification phase, place of cultivation and the type of *T. indica* [29].

The vitamins with the highest concentrations in the *T. indica* pulp are thiamine (0.34 mg), niacin (1.31 mg), riboflavin (0.13 mg), and ascorbic acid (3.96 mg). The pulp has a high organic acid content, including malic acid, acetic acid, citric acid, oxalic acid, and especially tartaric acid (8–12%), which is responsible for giving the fruit its characteristic taste [30]. *T. indica* contains several amino acids, including essential amino acids, such as leucine, lysine, and valine, which are vital for tissue growth and repair. It also contains non-essential amino acids, mainly aspartic acid and glutamic acid. In addition, *T. indica* has fatty acids that are also important because they can regulate cholesterol levels and inflammatory processes. Linoleic acid, oleic acid, and palmitic acid are present in the fruit pulp, but the pulp is not characterized by high levels of fatty acids like the seeds [27].

The seeds of *T. indica* are rich in phytochemical compounds with health properties and are valuable sources of several important industrial products, such as polysaccharides, protein, oil, kernel powder, gum, and starch [25,31]. The polysaccharide from *T. indica* seeds (TSP) is used in the food [32], pharmaceutical, and cosmeceutical industries, serving as stabilizers and drug delivery agents. Structural characterization of TSP has shown that it is a galactoxyloglucan composed of glucose, xylose, and galactose with a molar ratio of 3.1:1.7:1.0, respectively [33]. This polysaccharide has anti-inflammatory and probiotic effects, and it is also effective in tissue healing and regeneration by modulating different biochemical processes [34].

Regarding the phytochemical composition of *T. indica*, many authors have identified the presence of a wide range of bioactive molecules (Table 3). Of these, alkaloids, flavonoids, saponins, tannins, and terpenoids are the most frequently identified phytochemical compounds, not only in the fruit pulp but also in the seeds.

Polyphenols are the most studied phytochemical compounds in this fruit (Table 4). These polyphenols are mainly flavonoids, such as catechin and proanthocyanidins [44]. In addition to phenolic compounds and flavonoids, the chemical compositions of *T. indica* and their relative abundance are detailed in Table 4.

Moreover, Nurhanani et al. state that methanol is the most suitable solvent for obtaining the highest concentrations of phenolic compounds from different parts of *T. indica*, as well as obtaining the extract with the highest antioxidant activity in vitro [49]. Hossain et al. evaluated the influence of storage on the polyphenol count and antioxidant capacity of ethanolic extracts of *T. indica* pulp at different maturities and with different flavors. After 90 days of refrigerated storage, the authors reported a significant decrease in the polyphenol and flavonoid concentrations (around 50–70%) and in the 2,2-diphenyl-1-picrylhydrazyl (DPPH) scavenging activity (around 40–70%) of samples compared to their original state [48]. However, a recent study showed that packaging *T. indica* fruit in metallized polyester polyethylene laminate and storing it at 4 °C for a period of 180 days kept ascorbic acid content stable and lowered total soluble solids. On the other hand, total sugar values decreased with storage time, while titratable acidity and reducing sugars increased [50]. The polyphenols contribute to the anti-inflammatory and antioxidant effects of *T. indica*. This is due to their free radical scavenging activity, enhanced antioxidant and detoxification enzyme activity, and other biological functions. Flavonoids have been shown to have potent antidiabetic, cardioprotective, anti-inflammatory, and anticancer effects. They can exert their benefits through various pathways, both separately and in combination [51,52].

In addition to phenolic compounds and flavonoids, the chemical composition of *T. indica* and its relative abundance are detailed in Table 5.

*T. indica* also contains alkaloids, which are important compounds with therapeutic applications. These molecules have anti-inflammatory, antimicrobial, and anticancer properties and exert positive effects on neurodegenerative disorders [57,58]. Finally, triterpenoid saponins are present in tamarind. Phytochemical sources of saponins have come into focus due to increasing evidence of their health benefits, including their anti-inflammatory, immunostimulant, and hypoglycemic properties [59,60].

## 3. Therapeutic Effects of *Tamarindus indica*

*T. indica* has been used in traditional medicine in different regions for a long time, according to various reports. For example, Havinga et al.’s research on the uses of this tree in traditional African medicine reported that *T. indica* is mainly utilized as a laxative and for the treatment of wounds and abdominal pains, followed by diarrhea, helminth infections, fever, malaria, respiratory problems, and dysentery, as well as an aphrodisiac [61]. In addition, it is employed in Indian traditional medicine for the treatment of joint pain, inflammation, bronchial asthma, wound healing, burns, ocular diseases, colds, arthritis, dysuria, blood clots, diarrhea, dysentery, tonsillitis, dental diseases, and peptic ulcers [22].

There are several scientific studies on the therapeutic potential of *T. indica* (Table 6). These studies have demonstrated important properties of different parts of the plant (fruit, seeds, and leaves) using in vitro, in vivo, and in silico models. Some of their positive therapeutic properties are antibacterial [62], antioxidant [15], anti-inflammatory [16], cardioprotective [18], antidiabetic [17], hepatoprotective [63], ulcer-healing [64], and hypolipidemic [65] effects. The therapeutic mechanisms associated with *T. indica* are linked to its antioxidant, anti-inflammatory, and antimicrobial properties. Furthermore, clinical trials have been conducted with this fruit to evaluate its potential action [66,67].

### 3.1. Hepatoprotective Activities

The liver is a crucial organ in the human body that performs many physiological processes, including lipid, carbohydrate, and protein homeostasis; nutrient metabolism; detoxification; regulation of the immune system; and others [82]. Hepatocytes are the basic functional units of the liver, performing most of its functions [83]. In recent years, it has been estimated that more than two million people die each year worldwide from causes related to liver diseases. The most dangerous liver diseases are cirrhosis and hepatocarcinoma due to their high mortality rates [84]. In 2021, the “global incidence of cirrhosis and other chronic liver diseases was estimated to be approximately 58 million cases, with an age-standardized incidence rate of 724 per 100,000 persons, 1.4 million deaths, and 46 million disability-adjusted life-years” [85]. Furthermore, approved pharmacological treatment options for these conditions are limited [86]. Preserving the appropriate integrity of hepatocytes is key to normal liver function and is essential for human health [87].

**Figure 2:**
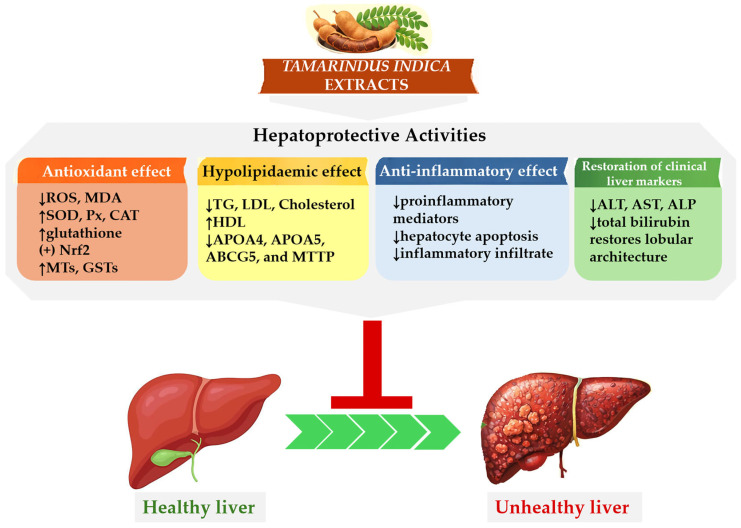
Diagram of hepatoprotective mechanism of *Tamarindus indica*. ↓: reduction or worsen, ↑: increase or improve, +: activation, MDA: malondialdehyde, TG: triglycerides, SOD: superoxide dismutase, CAT: catalase, Px: peroxidase, ROS: reactive oxygen species, Nrf2: nuclear factor erythroid 2-related factor 2, GSTs: glutathione S-transferases, MTs: metallothioneins, HDL: high-density lipoprotein, LDL: low-density lipoprotein, APOA: apolipoprotein A, ABCG5: ATP-binding cassette subfamily G member 5, MTTP: microsomal triglyceride transfer protein, ALT: alanine aminotransferase, AST: aspartate aminotransferase, ALP: alkaline phosphatase.

Much research on *T. indica* has focused on evaluating its hepatoprotective properties in liver diseases (Figure 2). In the fruit, some phenolic compounds have been identified as being capable of modulating inflammatory processes and preventing low-density lipoprotein (LDL) oxidation [44]. For example, Razali et al. showed that in HeGp2 cells, *T. indica* pulp extract upregulated genes involved in antioxidant response (metallothioneins and glutathione S-transferases) and downregulated those linked to hypolipidaemic effects (APOA4, APOA5, ABCG5, and MTTP) [68]. An in vivo study on rats by Lim et al. also found that fruit pulp increased hepatic antioxidant enzymes and regulated the hepatic gene expression of LDL receptor, ABCG5, APOA1, MTTP, and HMG-CoA reductase [47]. Upregulation of NRF2 and heme-oxygenase-1 at the protein and mRNA levels has also been reported in cells and animal models of liver injury induced by ethanol [63]. In another study, the presence of active polyphenolic compounds in the *T. indica* seed extract was identified as being capable of protecting hepatocytes against lipid peroxidation by acting as radical scavengers and reducing agents and by enhancing endogenous antioxidant activity [88]. Meena et al. [79] reported in their study that *T. indica* extract showed hepatoprotective activity against drug-induced hepatotoxic damage in rats. They observed a significant restoration of biochemical markers of liver damage. Also using a rat model, Yusuf et al. described that the *T. indica* extract was hepatoprotective due to anti-lipid peroxidative, antiapoptotic, and anti-inflammatory activities against aluminum hepatotoxicity [19].

### 3.2. Cardioprotective Activities

CVDs are the leading cause of death around the world, with more than 19 million deaths in 2023. Ischemic stroke, hypertensive heart disease, intracerebral hemorrhage, and ischemic heart disease were the leading causes of CVD-related mortality globally in 2023 [89]. Despite the new improvements in therapeutic options, CVD morbidity and mortality continue to rise [90], underscoring the need for novel therapeutic strategies targeting molecular pathways. Intake of a diet rich in polyphenols has been associated with a lower risk of CVD [91].

Bioactive compounds of *T. indica* exhibit cardioprotective effects, according to different research studies. In 2006, Martinello et al. suggested a potential anti-atherosclerotic effect of the *T. indica* extract [92]. They demonstrated that the sample was able to inhibit atherogenesis in the aorta of animals and improve blood cholesterol and triglyceride levels. At the same time, a human study reported similar improvements in the lipid profile through the action of this fruit. However, they did not find any effect on systolic blood pressure and body weight [93]. The hypolipidemic effects of *T. indica* have been associated with improved levels of cardioprotective proteins, such as serum antithrombin III and apolipoprotein A1 [65]. A combined in vivo and in silico study by Akter et al. demonstrated a significant reduction in the CVD biochemical markers evaluated (aspartate transaminase, C-reactive protein, lactate dehydrogenase, serum troponin I, creatinine kinase-MB, and lipid profiles) following treatment with the fruit extract, with the authors linking this effect to specific metabolites (2,3-dihydro-3,5-dihydroxy-6-methyl, 4H-pyran-4-one, and thymine) [45]. Another in vivo evaluation of the antihypertensive effects of ripened *T. indica* fruit extract found similar effects on the CVD markers evaluated. The molecular simulation test in this study identified gamma-sitosterol as the *T. indica* biometabolite with the best capacity to modulate and reduce hypertension and related risk factors (NR3C1, REN, guanylate cyclase receptor, PPARG, and CYP11B1) [42]. Moreover, Nisa et al. demonstrated that *T. indica* pulp nanoparticles showed promising cardioprotective effects (enhancing physiological redox balance and lipid metabolism and inhibiting apoptosis) against a rat model of induced cardiomyopathy [18]. Furthermore, clinical trials have also been performed, such as a study that evaluated the effects of *T. indica* fruit juice on the cardiometabolic health of patients living with HIV [66]. The study reported a potentially beneficial effect of *T. indica* on triglyceride metabolism and blood pressure homeostasis, but the results were not as conclusive as those found by Asgary et al. [67].

### 3.3. Antibacterial Activities

Bacterial infection and antibiotic resistance have become major health problems around the world [94]. Studies estimate that antimicrobial resistance could cause around 10 million deaths globally by 2050 [95]. Several phytopharmaceutical compounds have shown good antibacterial activity with lower toxicity [96,97]. *T. indica* extracts have shown promising antibacterial activity against *Salmonella*, *Enterococcus*, *Klebsiella*, *Shigella*, *Escherichia*, *Bacillus*, *Staphylococcus*, and *Pseudomonas* species, including different multidrug-resistant bacteria (Table 7). The antimicrobial effect of *T. indica* is attributed to the ability of its main bioactive compounds to disrupt the permeability of the microbial cytoplasmic membrane, triggering bacterial lysis. Additionally, these compounds may have direct effects on DNA and protein synthesis, interfering with bacterial metabolism, intercellular communication, and production of protease, lipase, and biofilm [14,98]. Synergistic interactions between these extracts and commonly used antibiotics have also been reported [14].

### 3.4. Antidiabetic Activities

Diabetes mellitus (DM) represents a major chronic disease worldwide, causing more than 3.4 million deaths in 2024 alone [103]. Its pathology is characterized by chronic hyperglycemia, often leading to cardiovascular disease, nephropathy, and neuropathy. The current study highlights the potential of phytochemical products in managing hyperglycemia and its complications [104].

**Figure 3:**
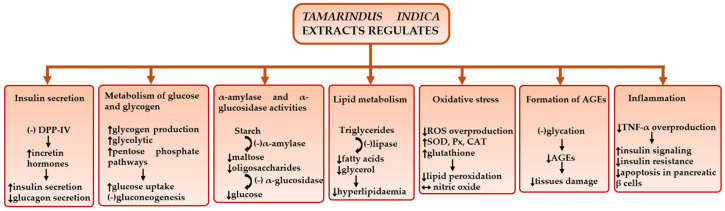
Diagram of the therapeutic mechanism of *Tamarindus indica* against diabetes mellitus and its complications. ↓: reduction or worsen, ↑: increase or improve, -: inhibition, ↔: normalizes, DPP-IV: dipeptidyl peptidase IV, SOD: superoxide dismutase, CAT: catalase, Px: peroxidase, TNF α: tumor necrosis factor-alpha, ROS: reactive oxygen species, AGEs: advanced glycation end products.

In vitro and in vivo studies have demonstrated the therapeutic effects of different parts of the *T. indica* tree against DM and its associated complications (Figure 3). These hypoglycemic effects can be linked to its multiple properties, such as antioxidant and anti-inflammatory effects, regulation of insulin secretion, and improvements in glucose and lipid metabolism [105]. Kathirvel et al. and Ouédraogo et al. conducted in vitro studies and found that *T. indica* (leaf, pulp, and seed extracts) reduced the activities of α-amylase and α-glucosidase [17,106]. Similar findings were reported by Krishna et al. (2020), who also observed an increase in glucose uptake [73]. Inhibition of α-glucosidase and α-amylase enzymes promotes a more sustained and controlled increase in blood glucose levels by suppressing starch digestion and delaying glucose absorption [107]. In addition, different in vivo animal studies have shown the ability of *T. indica* extracts to reduce blood glucose levels and increase blood insulin levels [76,108,109]. The antioxidant and anti-inflammatory activities are additional mechanisms of *T. indica* that may contribute to DM management.

### 3.5. Antioxidant Activities

In organisms, oxidative stress occurs as a result of an imbalance between reactive oxygen and nitrogen species production and the antioxidant defense system [110]. Chronic oxidative stress causes modifications in the functional and structural properties of important biomolecules (lipids, proteins, and DNA), contributing to the development of several diseases [111]. Both phenolic and non-phenolic compounds present in many plants are closely linked to beneficial antioxidant effects in human health [112]. In addition, there is a positive correlation between the polyphenolic content and antioxidant effect [113]. In vitro studies of *T. indica* antioxidant activities have shown a strong capacity to scavenge 2,2′-azino-bis(3-ethylbenzothiazoline-6-sulfonic acid) (ABTS), superoxide anion radical, DPPH, and nitric oxide [106,114]. Moreover, the plant extracts can help to improve the antioxidant defense system by increasing glutathione levels and enzymatic antioxidant activities, such as glutathione s-transferase, peroxidase, superoxide dismutase, and catalase [76,92,109,115]. Nisa et al. found that pulp aqueous extract derived from *T. indica* enhanced plasma antioxidant capacity and protected against lipid peroxidation damage in vivo [18]. The relevance of this aspect lies in the fact that oxidative stress is both a cause and a consequence of many chronic diseases, creating a harmful cycle that perpetuates itself [111].

### 3.6. Anti-Inflammatory Activities

Inflammation is a complex and essential component of the immune response [116]. However, at the same time, it is a contributing factor in the physiopathology of many chronic and non-chronic diseases, such as bronchial asthma, arthritis, metabolic syndrome, CVD, diabetes mellitus, COVID-19, cancer, and hepatitis [117]. Consumption of vegetables and fruits is associated with reduced risk of inflammation-related diseases [118].

Some in vitro and in vivo studies have demonstrated the potential beneficial effect of *T. indica* against inflammation-related diseases [119]. The methanolic extract of *T. indica* seeds administered on an induced edema rat model showed an anti-inflammatory and analgesic effect through reductions in edema volume and decreased levels of hematological parameters of inflammation, such as erythrocyte sedimentation rate, lymphocytes, and neutrophils [120]. Evaluation of a formulation of *T. indica* seed extract in a randomized clinical trial with 90 osteoarthritis subjects for 56 days showed that the serum concentrations of pro-inflammatory mediators (tumor necrosis factor-alpha (TNF α), interleukin (IL)-6, matrix metalloproteinase 3 (MMP3), and high-sensitivity C-reactive protein) were significantly reduced in the treated groups [121]. Sundaram et al. reported a reduction in the levels of inflammatory mediators, such as IL-1β, TNF-α, IL-6, IL-23, and cyclooxygenase-2, following oral administration of *T. indica* extract [53].

The capacity of *T. indica* fruit pulp extract to inhibit nitric oxide production and inducible nitric oxide synthase expression was found in an in vitro study using lipopolysaccharide-activated macrophages. Excessive production of nitric oxide is associated with many inflammatory diseases, including cancer [75]. Lima et al.’s study demonstrated a reduction in inflammatory infiltrate in the perirenal adipose tissue of Wistar rats treated with a trypsin inhibitor purified from *T. indica* seeds at 730 μg/kg for 10 days [122].

## 4. Bioavailability and Toxicology Studies

An in silico oral bioavailability study on the pulp and seed aqueous extracts of *T. indica* indicated good oral bioavailability of the studied bioactive compounds [45]. Toxicological evaluations are also important for the development of medical alternatives. Table 8 shows some examples of these studies for *T. indica*. An in vivo animal study on acute and chronic toxicity of *T. indica* fruit extract found the substance to be non-toxic at the tested dose [123]. Abubakar et al. reported that the “lethal dose 50% is greater than 5000 mg/kg body weight and can be classified as practically non-toxic and considered safe according to the recommendations of the WHO” [124].

The diversity and number of studies on the therapeutic effects of *T. indica* highlight the scientific community’s interest in its therapeutic potential. However, as in most cases when researching the therapeutic properties of natural products, it is necessary to evaluate factors such as the conditions under which the extracts are obtained, the bioavailability of bioactive compounds, and the structure–function relationship, among others. A study conducted by Loganathan et al. [72] involved a comprehensive evaluation of *T. indica* seed oil, beginning with the identification of the extraction method with the highest oil yield and optimization of the extraction process. In this research, the in silico analysis of plant-derived compounds included pharmacokinetic characteristics, drug-likeness properties, and molecular docking studies.

## 5. Conclusions

Effective medications for treating some pathologies, such as liver diseases, resistant bacterial infections, and diabetes, are limited. In the search for new treatments, natural alternatives may play a significant role in the prevention and complementary treatment of these pathologies. The evidence reviewed suggests that some *T. indica* compounds exhibit antioxidant, antimicrobial, anti-inflammatory, and anti-apoptotic activities. Several studies on *T. indica* have shown therapeutic effects and low toxicity in experimental models. Hydroalcoholic and aqueous extracts from the pulp and seeds of *T. indica* are recommended for diabetes, cardiovascular diseases, and liver diseases, with a dosage of between 100 and 500 mg/kg of body weight for 14 or 30 days for the best results. Meanwhile, hydroalcoholic extracts from the leaves are considered to have the best antibacterial effect. However, the heterogeneity of the extracts used in these studies creates a bias regarding the composition, dosage, and treatment regimen. In this regard, standardization of the active ingredients could provide greater reliability for their use as therapeutic candidates. The literature review did not find many studies evaluating the stability and bioavailability of the active compounds. This is an essential aspect for the development of future formulations and the use of technologies such as nanoencapsulation and microencapsulation. However, the mechanisms of action underlying the effects of *T. indica* on the evaluated pathologies still need to be clarified. Technologies such as multi-omics and bioinformatics can provide more information on the molecular signaling mechanisms involved. Although many of the studies analyzed in this review provide significant information on the therapeutic value of this plant, the possibility of establishing firm conclusions about its efficacy or toxicity is limited. Future research, particularly evaluations in chip or organoid models or controlled clinical trials, could reinforce the results obtained and support the future therapeutic use of *T. indica*. Overall, the results suggest that *T. indica* is a promising alternative that could contribute to the comprehensive treatment of different diseases.

## Figures and Tables

**Table 1:** Proximate composition of 100 g of *Tamarindus indica*.

	*T. indica* Pulp	*T. indica* Seeds
Carbohydrates	60.99 g	55.08 g
Protein	4.29 g	20.21 g
Total fibers	4.98 g	8.22 g
Lipid	1.36 g	7.23 g
Raw ash	3.48 g	3.42 g
Water	23.79 g	9.12 g

**Table 2:** Mineral composition of 100 g of *Tamarindus indica*.

	*T. indica* Pulp	*T. indica* Seeds
Potassium	318.56 mg	488.01 mg
Calcium	131.63 mg	108.08 mg
Phosphorus	70.4 mg	76.7 mg
Magnesium	38.58 mg	89.29 mg
Iron	3.95 mg	27.87 mg
Copper	5.77 mg	3.37 mg
Manganese	6.78 mg	3.1 mg
Sodium	38.21 mg	14.09 mg
Zinc	1.42 mg	3.31 mg

EtOH: ethanol, MeOH: methanol, +: present, -: absent, ND: not determined.

* Concentration of sample required to scavenge 50% of DPPH (µg/mL), TPC: total phenolic content, TFC: total flavonoid content, DPPH: 2,2-diphenyl-1-picrylhydrazyl, ABTS: 2,2′-azino-bis(3-ethylbenzothiazoline-6-sulfonic acid), GAE: gallic acid equivalent, RE: rutin equivalent, QE: quercetin equivalent, TE: Trolox equivalent, EtOH: ethanol, MeOH: methanol, ND: not determined.

**Table 3:** *Tamarindus indica*: phytochemical compounds.

Sample	Solvent	Alkaloid	Flavonoid	Glycoside	Saponin	Steroid	Tannins	Terpenoid	Ref.
Pulp	Water	+	+	+	+	-	+	-	[35]
	MeOH	+	+	+	+	-	+	-	
	Water	+	+	ND	+	ND	-	+	[36]
	EtOH	+	+	ND	+	ND	-	+	
	Water	+	-	+	+	ND	-	ND	[37]
	MeOH	+	-	+	+	ND	-	ND	
	Water	+	-	+	+	ND	-	ND	[38]
	EtOH	+	-	+	+	ND	-	ND	
	EtOH	-	+	ND	+	-	+	ND	[39]
	Water	+	+	+	+	-	+	+	[40]
	Water	+	+	ND	+	+	+	+	[41]
	Water	+	+	-	+	+	+	+	[42]
Seed	Water	-	+	-	+	ND	+	ND	[37]
	MeOH	+	+	-	+	ND	+	ND	
	Water	+	+	+	+	-	+	+	[40]
	Water	+	+	ND	+	ND	-	+	[36]
	EtOH	+	+	ND	+	ND	-	+	
	Ethyl acetate	-	+	-	+	-	-	+	[43]

EtOH: ethanol, MeOH: methanol, +: present, -: absent, ND: not determined.

**Table 4:** *Tamarindus indica* phenolic compounds and antioxidant characterization.

Sample	TPC	TFC	DPPH	ABTS	Ref.
*T. indica* flesh of sweet fruit pulp MeOH extract	99.70 ± 6.45 mg RE/g	397.53 ± 29.44 mg GAE/g	28.57 ± 1.04% *	ND	[45]
nanoparticles of *T. indica* water pulp extract	172.28 ± 1.23 mg GAE/g	134.53 ± 1.03 mg QE/g	70.18 ± 0.15%	86.48 ± 0.72%	[18]
*T. indica* cell suspension cultures MeOH extracts	71.73 mg GAE/g	ND	5.82 mmol TE/g	6.09 mmol TE/g	[46]
*T. indica* fruit pulp EtOH extract	244.96 ± 10.1 mg GAE/g	93.96 ± 2.6 mg RE/g	0.08 ± 0.01 mmol TE/g	0.07 ± 0.04 mmol TE/g	[47]
*T. indica* fruit pulp EtOH extract	40.57 ± 0.36 mg GAE/g	11.00 ± 0.17 mg QE/g	64.13 ± 1.05%	ND	[48]

* Concentration of sample required to scavenge 50% of DPPH (µg/mL), TPC: total phenolic content, TFC: total flavonoid content, DPPH: 2,2-diphenyl-1-picrylhydrazyl, ABTS: 2,2′-azino-bis(3-ethylbenzothiazoline-6-sulfonic acid), GAE: gallic acid equivalent, RE: rutin equivalent, QE: quercetin equivalent, TE: Trolox equivalent, EtOH: ethanol, MeOH: methanol, ND: not determined.

**Table 5:** Structure of selected bioactive compounds related to the hypoglycemic effects of *Tamarindus indica*.

Name/PubChem CID	Structure	Classification	Plant Part	Ref.
Catechin (CID 9064)	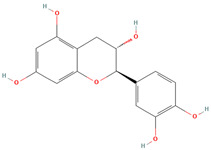	flavonoid	shell, fruit pulp, seed, leaf	[15,47,48,53,54]
Epicatechin (CID 72276)	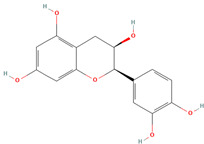	flavonoid	shell, fruit pulp, seed, leaf	[15,47,48,53,54]
Procyanidin B2 (CID 122738)	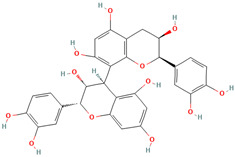	flavonoid	seed, leaf	[49,53]
Naringenin (CID 439246)	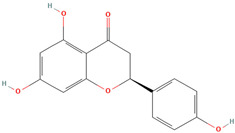	flavonoid	seed, leaf, shell, fruit pulp	[15,28,54]
Luteolin (CID 5280445)	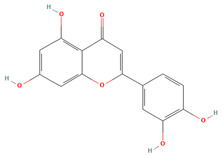	flavonoid	seed, shell	[15,44]
Gamma-Sitosterol (CID 457801)	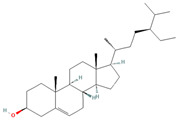	steroid	pulp, seeds	[42]
Taxifolin (CID 439533)	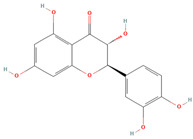	flavonoid	shell, seed	[15,44]
Oleic acid (CID 445639)	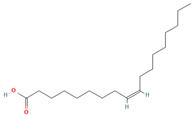	fatty acid	leaf	[19,55]
Cis-Vaccenic acid (CID 5282761)	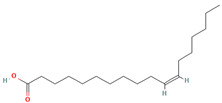	fatty acid	leaf, fruit pulp	[28,55]
5-(Hydroxymethyl)furfural (CID 237332)	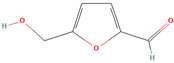	glycoside	fruit pulp	[56]
3-O-Methylglucose (CID 8973)	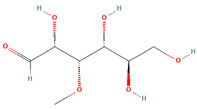	glycoside	fruit pulp	[56]
Eriodictyol (CID 440735)	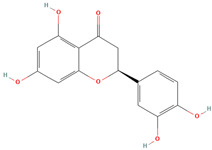	flavonoid	shell	[15]
Rutin (CID 5280805)	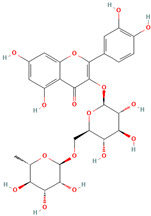	flavonoid	seed	[53]
Linoleic acid (CID 5280450)	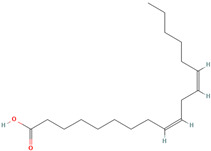	fatty acid	fruit pulp	[28]
Thymine (CID 1135)	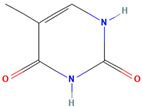	nucleotide base	fruit pulp, seed	[45]
4H-Pyran-4-one, 2,3-dihydro-3,5-dihydroxy-6-methyl (CID 119838)	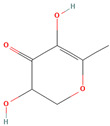		fruit pulp, seed	[45]
Quercetin (CID 5280343)	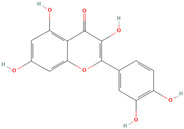	flavonoid	fruit pulp	[28]
Bis (2-ethylhexyl) phthalate (CID 16213881)	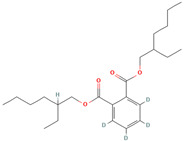	phenolic acid	fruit pulp	[14]
Phenol, 2,4-bis (1,1-dimethylethyl) (CID 7311)	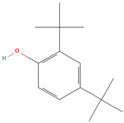	phenolic acid	fruit pulp	[14]
Bis(8-methylnonyl) (~2~H_4_)benzene-1,2-dicarboxylate (CID 71316284)	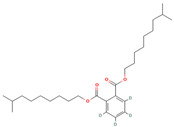	phenolic acid	fruit pulp	[14]
Hesperidin	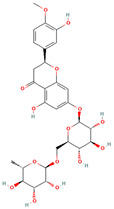	flavonoid	fruit pulp	[28]
Pyrogallol (CID 1057)	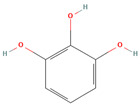	phenolic acid	fruit pulp	[28]
Apigenin (CID 5280443)	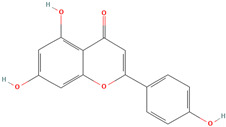	flavonoid	Shell, seed	[15,44]

CID: compound identifier.

**Table 6:** Therapeutic evaluation of *Tamarindus indica*.

Study	Sample	Active Principle	Model	Main Results	Ref.
antimicrobial activities against drug-resistant bacterial strains	*T. indica* fruit EtOH extract	bis (2-ethylhexyl) phthalate; phenol,2,4-bis(1,1-dimethylethyl); 1,2-benzenedicarboxylic acid; bis(8-methylnonyl) ester	in vitro in silico	antimicrobial activities against *Pseudomonas aeruginosa* and *Staphylococcus aureus*	[14]
antihypertensive effects against cholesterol-induced hypertensive	*T. indica* pulp and seed aqueous extracts (50 mg/kg BW and 100 mg/kg BW)	gamma-sitosterol	in vivo in silico	dose-dependent antihypertensive effect	[42]
hepatoprotective effects against ethanol-induced toxicity	*T. indica* shell EtOH extract 750 µg/egg	ND	in vitro in vivo	↓ ethanol-induced oxidative stress via + Nrf2 that improves ALD	[63]
gene expression profiles in human HepG2 cells against *T. indica* extract	*T. indica* fruit MeOH extract 300 µg/mL	ND	in vitro	↑ gene expression to stress response (MT and GTS) and hypolipidemic action via ↓ gene expression of lipid metabolic proteins	[68]
cardioprotective effects in doxorubicin-induced cardiotoxicity	*T. indica* pulp and seed aqueous extracts 200 mg/kg	thymine; 4H-pyran-4-one, 2,3-dihydro-3,5-dihydroxy-6-methyl-	in vitro in vivo in silico	antioxidative agent and prevent cardiotoxicity	[45]
anti-arthritic effects against induced septic arthritis	*T. indica* leaves EtOH extract 1000 mg/kg	ND	in vitro in vivo	↓ synovial LDH level, anti-arthritic effects and promising antimicrobial	[69]
antitumor and Immunopotentiating Activity	*T. indica* seeds 200 mg/kg BW	polysaccharide	in vitro in vivo	promising anticancer agent with immunomodulatory action	[70]
regulating the composition of gut microbiota	*T. indica* seeds	polysaccharide	in vitro	anti-inflammation and prebiotic effects preserves the intestinal epithelial barrier	[71]
ameliorating induced arthritis	*T. indica* seeds EtOH extract 50 mg/kg	threo-isocitric acid; galactosyl glycerol; procyanidin B2; arecatannin B1; catechin; rutin; embelin	in vivo	↓ stress oxidative, inflammation and cartilage/bone degradation	[53]
reducing LDL cholesterol	*T. indica* seeds petroleum ether extract	cycloartal and campesterol	in silico	promising (-) LDL receptor protein	[72]
anti-inflammatory and wound healing activities	*T. indica* bark, seeds, fruit and leaves n-hexane extracts 100 μg/mL	lupeol; n- docosanoic acid; methyl tricosanoate; α-terpinyl acetate; lupeol acetate	in vitro in silico	wound healing and anti-inflammatory properties	[16]
antihyperglycemic activity	*T. indica* pulp aqueous extracts	glycosides, alkaloids and anthraquinones	in vitro	(-) enzymes such as α-amylase and α-glucosidase enhanced glucose absorption potential	[73]
hepatoprotective potential against aluminum exposure	*T. indica* leaves ethyl acetate extract 800 mg/kg	oleic acid; phenol, 3,5-bis (1,1-dimethyl ethyl); n-hexadecanoic acid	in vivo	antiapoptotic, ↓ lipid peroxidative and ↓ inflammation	[19]
anti-inflammatory, antidiabetic, and biocompatibility properties	*T. indica* fruit coat aqueous extract 30 mg/mL	ND	in vitro in vivo	dose-dependent anti-inflammatory and antidiabetic effect	[17]
anti-angiogenic Activity	*T. indica* pericarp and seeds MeOH extracts	catechin, naringenin and procyanidin B	in vitro in silico	(-) cell viability by ↓ angiogenesis (via (-) VEGF)	[54]
cardioprotective effects in doxorubicin-induced cardiotoxicity	MgO nanoparticles of *T. indica* pulp aqueous extract 30 mg/kg	polyphenols	in vitro in vivo	cardioprotective, hypolipidemic, antioxidant, and antiapoptotic	[18]
anti-proliferative effects in colorectal cancer cells	*T. indica* seeds MeOH extract	gallic acid, ferulic acid, and naringenin	in vitro	promote apoptosis ↑ ROS production (+) pro-apoptotic proteins ↓ NF-*κ*B activity cell cycle arrest	[74]
hypolipidemic effects in diet-induced hypercholesterolemic	*T. indica* pulp EtOH extract 500 mg/kg	ND	in vivo	↔ lipid profile and levels of cardioprotective serum proteins (APOA1, ATIII, TF and VDBP)	[65]
anti-inflammatory effect in LPS-stimulated macrophages	*T. indica* pulp aqueous extract	ND	in vitro	↓ inflammation, (-) production of nitric oxide and gene expression of iNOS	[75]
antidiabetic and antioxidative activities	*T. indica* seed n-hexane fraction of hydro- MeOH extract of 100 mg/kg	flavonoids, alkaloids, terpenoids and steroids, while saponins, glycosides, tannins, protein, anthraquinones and phlobatannins	in vitro in vivo	prevent hyperglycemia, improve plasma insulin and C-peptide levels ↑ antioxidant enzymes activities	[76]
antioxidant activity	*T. indica* cell suspension MeOH extract	naringenin, catechin, procyanidin, kaempferol, eriodictyol, and atrovenetinone	in vitro	high phenolic content strong antioxidant activity	[46]
anti-ulcerative colitis effect	*T. indica* pulp and seed EtOH extract 500 mg/kg	ND	in vivo	improve colon weight, ulcer indices, and total colitis ↓ MPO activity and MDA levels	[64]
anticancer action	*T. indica* seed dichloromethane, ethyl acetate, and n-butanol extract 47.3 g/mL	ND	in vitro	cytotoxic effect via interaction with the reduced glutathione cycle and antioxidant enzymes	[77]
cardioprotective activity against Doxorubicin-induced cardiotoxicity	*T. indica* leaves EtOH extract 60 μg/mL	tannins and flavonoids	in vitro in vivo	↑ cardiac output, force of contraction, and heart rate ↓ serum levels of CPK and LDH	[78]
antioxidant potential	*T. indica* shell EtOH extract (50, 100, 200 µg/mL) (5, 10, 20 µg/mL)	catechin, taxifolin, myricetin, eriodictyol, luteolin, morin, apigenin, naringenin	in vitro in vivo	free radical scavenging activity ↓ MDA and ROS levels ↔ SOD activity	[15]
hepatoprotective activity against antitubercular drugs, hepatotoxicity	*T. indica* stem bark EtOH extract 200 mg/kg BW	ND	in vivo	↔ liver-specific enzymes and biochemical markers altered	[79]
antitumor Efficacy	*T. indica* seeds	chitosan–T. indica gum polysaccharide	in vitro	↑ control over the cancer cell growth	[80]
antioxidant activities and potential hypocholesterolaemic properties	*T. indica* pulp EtOH extract 500 mg/kg BW	polyphenols and flavonoids (Catechin)	in vitro in vivo	↑ cholesterol efflux, LDL uptake and clearance ↓ triglyceride accumulation (-) cholesterol synthesis ↑ hepatic antioxidant enzymes	[47]
hepatoprotective effect against antitubercular drugs hepatotoxicity	*T. indica* fruit aqueous extract 500 mg/kg	ND	in vivo	↓ levels of hepatotoxic biomarkers ↔ hepatic antioxidant enzymes (Px, SOD, and CAT) activity	[81]

ALD: alcohol liver disease, Nrf2: nuclear factor erythroid 2-related factor 2, LDH: lactate dehydrogenase, LDL: low-density lipoprotein, VEGF: vascular endothelial growth factor, EtOH: ethanol, MeOH: methanol, APOA1: apolipoprotein A1, ATIII: antithrombin III, SOD: superoxide dismutase, CAT: catalase, Px: peroxidase, ABCG5: ATP-binding cassette subfamily G member 5, MPO: myeloperoxidase, MDA: malondialdehyde, ROS: reactive oxygen species, LDH: lactate dehydrogenase, CPK: creatinine phosphokinase, iNOS: inducible nitric oxide synthase, ↓: reduction, ↑: increases, +: activation, -: inhibition, ↔: normalizes, ND: not determined, BW: body wide.

**Table 7:** Antibacterial evaluation of *Tamarindus indica*.

Sample	Active Principle	Tested Strains (MIC)	Result	Ref.
*T. indica* fruit pulp, acetone and EtOH extract	ND	*Klebsiella pneumoniae* (9.38 mg/mL) *S. aureus* (18.75 mg/mL)	both acetone and ethanol extracts showed significant antibacterial activities	[62]
*T. indica* fruit pulp, leaves and Stem bark water and EtOH extract	carbohydrates, reducing sugars, tannins, alkaloids, and saponins	*Bacillus subitili*, *Escherichia coli* (7.81–31.25 mg/mL)	fruit pulp extracts exhibited a wide spectrum of activity	[99]
*T. indica* leaves and fruit water and MeOH extract	alkaloid, glycoside, saponin, tannin, anthraquinone and steroid, reducing sugar, flavonoid, terpenoid and phenol	*E. coli* and *Shigella* sp. (3.125–25 mg/mL)	antimicrobial effect and higher activity in MeOH extract	[100]
*T. indica* fruit pulp MeOH extract 70%	5-Hydroxymethylfurfural (31.06%), -3-O-Methyl-d-glucose (16.31%), 1,6-anhydro-βD-Glucopyranose (9.95%)	*Plesiomonas shigelloides*, *Bacillus pumilus* and *E. coli* (0.22–1.76 mg/mL) *Bacillus cereus* and *Klebsiella pneumoniae* (3.51 mg/mL) *Acinetobacter calcaoceuticus* and *Staphylococcus aureus* (>7.02 mg/mL)	*P. shigellosis* and *B. pumillus* are more sensitive	[56]
*T. indica* leaves and fruit MeOH, EtOH, isopropanol and water extract	carbohydrates, flavonoids, vitamin C and alkaloids	*E. coli*, *Staphylococcus* sp., *Pseudomonas* sp., *Bacillus* sp. and *Klebsiella* sp.	leaves’ EtOH extract showed the best antibacterial activity, while the fruit isopropanol extract was the best activity	[101]
*T. indica* fruit EtOH extract 80%	bis(2-ethylhexyl)phthalate, phenol,2,4-bis(1,1-dimethylethyl), 1,2-benzenedicarboxylic acid, and bis(8-methylnonyl) ester	*S. aureus* (0.78, 3.12 mg/mL) *P. aeruginosa* (1.56, 3.12 mg/mL)	synergistic effects with conventional antibiotics (imipenem, amikacin, and ofloxacin) and reduced their MICs; the extract was able to influence cellular membrane permeability	[14]
*T. indica* leaves and fruits EtOH extract 80%	phenolic compounds	*S. aureus* and *P. aeruginosa* (500 µg/mL)	extracts have antimicrobial effects and are an antioxidant source	[102]

MIC: minimum inhibitory concentration, EtOH: ethanol, MeOH: methanol, ND: not determined.

**Table 8:** Toxicity and safety evaluations in *Tamarindus indica*.

Study	Sample	Model	Result	Ref.
acute oral toxicity and oral mucous irritability studies	*T. indica* fluid extract	rats and hamsters	classified as a non-toxic substance	[123]
heavy metal, aflatoxin, and pesticide residues (in vitro)	*T. indica* Fruit	-	harmful substances under the permissible limits	[125]
acute and sub-acute oral toxicity study	*T. indica* leaves	rabbit	*T. indica* leaves did not show any toxic or adverse effect under work concentrations (500 and 1000 mg/kg)	[69]
chronic toxicity study	*T. indica* pulp	wistar rat	after six months, pulp extract was generally safe and well-tolerated at the tested dose	[126]
acute oral toxicity study	*T. indica*	Wistar male rats	extract was framed as a non-toxic substance	[127]
acute toxicity study	*T. indica* fruits	zebrafish larvae	were non-toxic to both invertebrate models, with LC50 values substantially greater than 1000 μg/mL	[128]
acute oral toxicity study	*T. indica* pulp, leaves and stem bark	Wister albino rats	pulp extract (3000 mg/kg and 5000 mg/kg BW) resulted in no mortality	[124]
acute oral toxicity studies	*T. indica* trypsin inhibitor	Embryonic and adult zebrafish	trypsin inhibitor isolated from *T. indica* has biosafety and therapeutic potential	[129]

LC50: lethal concentration 50%.

## Data Availability

No new data were created or analyzed in this study.

## Abbreviations

ABCG5	ATP-binding cassette subfamily G member 5
ABTS	2,2′-azino-bis(3-ethylbenzothiazoline-6-sulfonic acid
ALD	alcohol liver disease
APOA1	apolipoprotein A1
APOA5	apolipoprotein A5
ATIII	antithrombin III
CAT	catalase
CID	compound pdentifier
COVID-19	coronavirus disease 2019
CPK	creatinine phosphokinase
CVD	cardiovascular diseases
DM	diabetes mellitus
DPPH	2,2-diphenyl-1-picrylhydrazyl
DPP-IV	dipeptidyl peptidase IV
ESR	erythrocyte sedimentation rate
EtOH	ethanol
GAE	gallic acid equivalent
GST	glutathione S-transferase
HIV	human immunodeficiency virus
HMG-CoA	hydroxymethylglutaroyl coenzyme A
IL	interleukin
iNOS	inducible nitric oxide synthase
LC50	lethal concentration 50%
LDH	lactate dehydrogenase
LDL	low-density lipoprotein
MDA	malondialdehyde
MeOH	methanol
MIC	minimum inhibitory concentration
MMP3	matrix metalloproteinase 3
mRNA	messenger RNA
MT	metallothionein
MTTP	microsomal triglyceride transfer protein
ND	not determined
NF-κB	nuclear factor-κB
NO	oxide nitric
Nrf2	nuclear factor erythroid 2-related factor 2
Px	peroxidase
QE	quercetin equivalent
RE	rutin equivalent
ROS	reactive oxygen species
SOD	superoxide dismutase
TE	Trolox equivalent
TFC	total flavonoid content
TNF α	tumor necrosis factor-alpha
TPC	total phenolic content
TSP	polysaccharide from *T. indica* seeds
WHO	World Health Organization
